# Transcriptomic profiling and experimental validation of myeloid-cell-differentiation-related key genes in osteoarthritis

**DOI:** 10.3389/fgene.2026.1820192

**Published:** 2026-06-17

**Authors:** Jiaming Liu, Xinyu Zhang, Hai Xu, Xueyi Fu, Duanyang Sheng, Yinghe Huang, Yuanxin Huang, Xianglong Lv, Wei Lu

**Affiliations:** 1 Department of Orthopedics, Beijing Jishuitan Hospital Guizhou Hospital, Guiyang, China; 2 Guizhou Medical University, Guiyang, China; 3 Department of Pain Medicine, Affiliated Hospital of Guizhou Medical University, Guiyang, China

**Keywords:** gene, machine-learning technique, myeloid cell differentiation, osteoarthritis, transcriptomics

## Abstract

**Objective:**

This study aims to leverage publicly available databases to systematically investigate the pathogenic mechanisms of osteoarthritis (OA), with particular focus on the role of myeloid cell differentiation (MCD)-related genes. Comprehensive multidimensional analyses were performed to elucidate the potential mechanisms through which these genes contribute to the pathophysiological processes of OA. The findings of this study are expected to provide a theoretical foundation for targeting MCD-related abnormalities in OA.

**Methods:**

We systematically integrated data acquisition, differential expression analysis, intersection with MCD-associated gene sets, machine learning-based feature selection, and multidimensional bioinformatics analysis—including functional enrichment, immune infiltration profiling (using the CIBERSORT algorithm), and structure-guided molecular docking to elucidate the molecular links between MCD and OA pathogenesis. We subsequently performed a comprehensive suite of functionally complementary downstream analyses, including nomogram construction for clinical risk prediction, receiver operating characteristic (ROC) curve analysis to assess diagnostic performance, decision curve analysis (DCA) to evaluate clinical utility, and structure-based molecular docking to probe potential ligand–target interactions.

**Results:**

We identified eight key genes (*GRP183*, *MFAP2*, *NDP*, *TF*, *TFRC*, *TYROBP*, *VEGFA*, and *ZBTB16*) through systematic screening. Following expression level validation, *VEGFA*, *ZBTB16*, and *TYROBP* were found to exhibit consistent expression trends and statistically significant differences across two independent datasets. Using the immunological atlas (the CIBERSORT algorithm), we estimated the infiltration levels of 22 immune cell subtypes and found that immune cell infiltration was significantly associated with OA progression and molecular subtype. Furthermore, molecular docking simulations were performed between the three key genes and two candidate therapeutic compounds, which provided preliminary insights and potential clues for future clinical drug development targeting these molecular interactions. To investigate the mRNA expression levels of the key genes, we performed real-time quantitative reverse transcription polymerase chain reaction (RT-qPCR) analysis.

**Conclusion:**

We integrated transcriptomic data with bioinformatics approaches and machine-learning techniques in this study to identify potential biomarkers associated with OA. We identified *VEGFA*, *ZBTB16*, and *TYROBP* as three key MCD-associated genes in OA; we further explored their biological functions and underlying regulatory mechanisms, which were supported by the experimental validation of the expression of the key genes.

## 1 Introduction

Osteoarthritis (OA) is a whole-joint disease characterized by meniscal degeneration alongside inflammation and fibrosis of the infrapatellar fat pad and synovium, all of which can significantly compromise quality of life (Dong et al., 2023). The pathogenesis of OA is multifactorial and complex, involving a range of contributing factors such as aging, obesity, genetic predisposition, joint trauma, inflammation, and metabolic dysregulation (Dong et al., 2023; Musumeci et al., 2015). The intrinsic capacity of the body to repair articular cartilage diminishes with age, making it increasingly susceptible to damage and degeneration (Ghouri et al., 2022). Some genetic factors also contribute to OA susceptibility, as specific gene mutations and polymorphisms have been linked to an increased risk of OA (Jiang, 2022). Inflammatory processes and metabolic disturbances are also implicated in OA progression; for example, following a joint injury, the synovial tissue and chondrocytes secrete pro-inflammatory cytokines, and the release of these pro-inflammatory mediators can promote cartilage destruction and synovial inflammation (Brophy and Fillingham, 2022). Although pharmacological interventions are effective in relieving pain and inflammation, they may be associated with adverse effects (Hodgkinson et al., 2022). Surgical interventions are often potentially beneficial for restoring joint function but involve risks, including infection, hemorrhage, thromboembolic events, and implant loosening (Thoene et al., 2023; Chen et al., 2023a). Currently, therapeutic strategies offer limited efficacy, which highlights the urgent need to identify novel molecular targets for intervention.

Myeloid cells constitute a major lineage of hematopoietic cells derived from hematopoietic stem cells and primarily include granulocytes, monocytes, macrophages, and dendritic cells (Christofides et al., 2023). Myeloid cell differentiation (MCD) is a tightly regulated biological process (BP) governed by a network of cytokines and transcription factors (Chen et al., 2022; Yamazaki et al., 2024). For example, granulocyte colony-stimulating factor (G-CSF) promotes granulopoiesis, whereas monocyte colony-stimulating factor (M-CSF) drives monocyte differentiation (Wu et al., 2024; Xu et al., 2023). Inflammation plays a central role in the pathogenesis of OA (Zhou et al., 2023; South et al., 2023); concurrently, these inflammatory mediators can induce synovial hyperplasia, leading to joint swelling and pain. As antigen-presenting cells within the myeloid lineage, dendritic cells activate T cells and B cells, thereby participating in the adaptive immune response associated with OA (Peña et al., 2022). Moreover, regulatory T cells and other immunomodulatory lymphocytes may interact with myeloid cells to modulate the inflammatory and immune responses in OA (Pang et al., 2023).

Among OA patients, certain myeloid cell subsets may also influence chondrocyte metabolism, thereby affecting cartilage repair and regeneration processes (Hubbard et al., 2020). Accumulating evidence from multiple studies has indicated that there is substantial myeloid cell infiltration of the synovium and subchondral bone in OA patients (Yamazaki et al., 2024). Notably, the number of macrophages is significantly elevated in the synovial tissues of OA patients and is correlated with disease severity (Von Kaeppler et al., 2021; Fang et al., 2024). In addition, synovial fibroblasts in OA can secrete factors, such as R-spondin 2, to regulate MCD and inflammatory responses, thereby contributing to further joint damage (Knights et al., 2023). To date, existing studies have examined only isolated associations between individual MCD-related genes (MCDRGs) and OA, and there has been no comprehensive screening of the MCD-associated gene network nor any mechanistic investigation of their functional interplay. In contrast, our study identifies a set of MCD-centered core genes through integrative bioinformatics and experimental validations; we further elucidate their synergistic regulatory roles in OA pathogenesis, thereby addressing a critical knowledge gap and offering novel mechanistic insights (Jiang et al., 2025). Further investigations into the interplay between MCD and OA may unveil novel therapeutic targets and strategies for effective management of OA. Current evidence on myeloid cell involvement in OA is fragmented and largely limited to macrophage polarization and single-gene studies without a systematic characterization of MCDRGs or their regulatory networks (Yin et al., 2024). Moreover, reliance on single-cohort analyses and limited experimental validations constrains the robustness and translational relevance of these studies (Zhang and Ji, 2023). Therefore, an integrative multicohort analysis with experimental confirmation is needed to define the coordinated roles of MCDRGs in OA.

The regulatory mechanisms underlying MCDRGs in OA remain poorly characterized, and there are no available clinical validations of MCD-associated biomarkers or therapeutic targets. Transcriptomic analysis can be used to elucidate the functional mechanisms of MCDRGs in OA, thereby enabling identification of key candidate genes that could serve as precise therapeutic targets. To address this gap, we integrated the least absolute shrinkage and selection operator (LASSO) algorithm with machine learning and transcriptomic profiling, multilayered bioinformatics analysis, and experimental validation to identify the key MCDRGs. The primary objective of this study was to establish a prioritized roadmap of MCDRGs in OA. By integrating cross-platform transcriptomic data with machine learning techniques, we aimed to distill the core regulatory hubs from among thousands of candidates to provide a robust bioinformatic foundation for subsequent bench-to-bedside functional validations.

## 2 Materials and methods

### 2.1 Bioinformatics analysis

#### 2.1.1 Data collection

First, OA-related transcriptomic datasets, including both the training and validation sets, were obtained from the Gene Expression Omnibus (GEO) database (https://www.ncbi.nlm.nih.gov/). All samples included in this study were derived from human synovial tissues. The training cohort (GSE89408) comprised 22 OA patients (mean age: 65.3 ± 8.2 years; male: female ratio = 9:13; Kellgren–Lawrence (K-L) grade III-IV, n = 18; K-L grade II, n = 4) and 28 normal controls (mean age: 62.5 ± 7.8 years; male: female ratio = 11:17; no history of joint disease); detailed information regarding the GSE89408 dataset is provided in Supplementary Table S1. The independent validation cohort (GSE55235) consisted of 10 OA patients (mean age: 64.7 ± 9.1 years; male: female ratio = 4:6; K-L grade III-IV, n = 8; K-L grade II, n = 2) and 10 healthy controls (mean age: 61.2 ± 8.5 years; male: female ratio = 5:5; no history of inflammatory joint disease or OA). The severity of OA was assessed using the K-L radiographic grading scale (Dong et al., 2023). Datasets were included if they contained synovial tissue samples with clearly defined OA and normal groups with complete gene expression data; samples lacking clear grouping information, derived from a non-synovial origin, and corresponding to rheumatoid arthritis were excluded from the analysis.The GSE89408 and GSE55235 datasets were analyzed independently as training and validation cohorts to avoid cross-dataset batch effects. The raw counts in GSE89408 were normalized via the trimmed mean of M values method and log_2_-transformed, while the data in GSE55235 were log_2_-transformed from the provided normalized matrix. As the individual datasets were processed under consistent conditions, no additional batch corrections (e.g., ComBat) were required.

#### 2.1.2 Differential expression analysis coupled with rigorous candidate gene prioritization

The MCDRGs were extracted from the Molecular Signatures Database (MSigDB; https://www.gsea-msigdb.org/; Wu et al., 2024) and specifically from the Gene Ontology (GO) BP gene set “GOBP_MYELOID_CELL_DIFFERENTIATION” (MSigDB ID: M11726; version 2023.2), which represents a curated functional gene set rather than a manually defined set in this study. A total of 444 genes were extracted from this gene set for subsequent analyses. All computational analyses were performed using R software (version 4.5.2) and Cytoscape (v3.9.4; https://cytoscape.org/). The functional and interaction data were retrieved from established peer-reviewed bioinformatics resources: STRING (v12.0; https://cn.string-db.org/), miRDB (v6.0; https://mirdb.org/), UniProt (https://www.uniprot.org/), and DSigDB (v1.0; https://dsigdb.tanlab.org/DSigDBv1.0/). All R packages used in this study, including edgeR (v4.0.10) for differential expression analysis, ggplot2 (v3.4.4) for publication-quality visualization, clusterProfiler (v4.8.0) for functional enrichment mapping, and glmnet (v4.1-8) for regularized regression modeling, were obtained from Bioconductor (https://www.bioconductor.org/), which is a rigorously curated and peer-reviewed repository for computational biology software.

The differential gene screening process was as follows: the genes were considered to be significantly differentially expressed at an adjusted *p*-value < 0.05 (Benjamini–Hochberg method) and a |log_2_ (fold change)| > 1. The protein–protein interaction (PPI) network construction (STRING v12.0, confidence mode) was as follows: PPIs with a combined score of >0.4 were retained to balance the sensitivity and specificity. For machine learning-based feature selection, LASSO regression was chosen due to its ability to perform variable selection and regularization simultaneously, thereby reducing model complexity and mitigating overfitting in high-dimensional transcriptomic data. The optimal lambda value was determined via five-fold cross-validation, and the lambda.min value (corresponding to the minimum cross-validated error) was selected to identify candidate feature genes with non-zero coefficients. Gene-set enrichment/verification analyses (GSEA/GSVA) were conducted as follows: gene-set enrichment was first performed using the MSigDB C2 canonical pathways collection (c2. cp.kegg.v7.5.1symbols.gmt), where significant enrichment was defined as a |normalized enrichment score (NES)| > 1 and an adjusted *p*-value < 0.05 (false discovery rate (FDR) correction). For immune infiltration analysis (CIBERSORT v1.0.6, LM22 signature matrix), only samples with a deconvolution *p*-value < 0.05 and a correlation coefficient >0.6 between the observed and predicted expression profiles were retained; then, immune cell proportions were estimated using the LM22 reference matrix (v2019.1). Finally, the candidate genes were identified as follows: ① The differentially expressed genes (DEGs) were first identified by comparing the transcriptomic profiles between the two experimental groups using established bioinformatic tools; here, we applied stringent statistical thresholds (|log_2_(FC)| ≥ 1 and adjusted *p*-value < 0.05) and visualized the resulting data using volcano plots and heatmaps. ② The high-confidence candidate genes were then rigorously prioritized by intersecting the list of DEGs with functionally enriched and biologically relevant gene sets, such as those associated with myeloid differentiation, immune regulation, or disease-relevant pathways, thereby focusing on genes with both differential expression and mechanistic plausibility. DEGs were then identified by comparing OA patients with control samples in the training set.

#### 2.1.3 Analysis of candidate gene functions and interactions

The candidate genes were screened by taking the intersection of DEGs and MCDRGs. Functional enrichment analyses (GO and KEGG) and PPI network construction were performed to explore the biological functions, signaling pathways, and molecular interactions associated with these candidate genes in the pathogenesis of OA.

#### 2.1.4 Screening and validation of key genes

To identify key regulatory genes, we applied machine learning algorithms to the candidate genes using the training set. Subsequently, the expression levels of the feature genes were evaluated using both the training and validation sets to finalize the list of key genes.

#### 2.1.5 Multidimensional bioinformatics analysis of key genes

The multidimensional analysis of the list of key genes involved several steps as detailed herein. ① Development and rigorous evaluation of diagnostic classifiers, which included construction of interpretable, machine-learning-based diagnostic models trained on the expression profiles of the key genes. ② Correlation analysis, where specific statistical methods were used to analyze expression correlations among the key genes and visualize them. ③ Construction of functional association networks, where public databases were utilized to construct co-expression networks of the key genes and explore the functionally associated genes. ④ Molecular regulatory network analysis, where regulatory molecules of key genes were predicted using databases and by constructing regulatory networks. Here, potential miRNA–mRNA regulatory mechanisms underlying the candidate biomarkers in OA were investigated via an integrative cross-database analysis using three experimentally supported and widely curated resources: miRDB (v6.0), miRTarBase (v8.0), and DIANA-TarBase (v8). Target miRNAs were included only when they were supported by two or more databases, where at least one experimental validation was documented in miRTarBase or DIANA-TarBase, thereby prioritizing biologically credible interactions over purely computational predictions. A high-confidence miRNA–mRNA regulatory network was then constructed in Cytoscape (v3.9.2), and the topological properties such as degree centrality, betweenness centrality, and module structure were quantitatively assessed and visualized. ⑤ Genomic and subcellular localization profiling, where the chromosomal coordinates were annotated, and high-confidence subcellular localization was inferred using consensus predictions from multiple tools. ⑥ Multitier pathway enrichment dissection was performed, entailing complementary pathway analyses, including single-gene-based overrepresentation (GO/KEGG), GSEA, and GSVA, to capture both categorical and continuous pathway activity differences associated with key genes. ⑦ Deconvolution-based immune infiltration quantification and association modeling, where immune cell abundances were estimated across bulk RNA-seq samples using CIBERSORT to identify significantly enriched/depleted immune subsets (adjusted *p value* < 0.05) and model their associations with key gene expression via multivariate linear regression or Spearman correlation. ⑧ Candidate compounds targeting the key genes were retrieved from the DSigDB database and prioritized based on combined scores, statistical significance, and multitarget potential. Here, the three-dimensional structures of the ligands were obtained from PubChem, while the protein structures were retrieved from the AlphaFold database and used without further energy minimization. Next, molecular docking was performed using the CB-Dock2 platform by integrating cavity detection with AutoDock Vina; the docking simulations were conducted with the default parameters, and the binding affinities were evaluated using Vina scores, where lower values were considered to indicate stronger predicted binding capacities. Complexes with binding energies ≤ −4.5 kcal/mol were considered to exhibit favorable binding potential and were selected for further analysis. The binding modes were further assessed based on interaction stability, including hydrogen bonding, hydrophobic contacts, and potential electrostatic interactions within the binding interface. To validate docking reliability, the native ligands were removed and redocked under identical conditions. The root mean-squared deviation (RMSD) between the predicted and native conformations was calculated, where RMSD values < 2.0 Å were considered as indicating acceptable accuracy.

### 2.2 Experimental verification

Synovial tissue was selected as the primary tissue for analysis because it serves as the principal site of immune cell infiltration, particularly myeloid cells, in OA; the inflammatory microenvironment of this tissue directly orchestrates MCD, aligning precisely with the central mechanistic focus of this study. Moreover, synovial tissue is routinely accessible during arthroplasty or arthroscopic procedures in OA patients, and its molecular profile robustly reflects the local joint inflammatory and immune-dysregulated state. In contrast, articular cartilage is difficult to procure without introducing mechanical or enzymatic damage, which renders the samples highly susceptible to RNA degradation and chondrocyte dedifferentiation; furthermore, subchondral bone exhibits markedly lower expression levels of MCD-associated genes, which limits its utility in detecting transcriptional signatures of myeloid lineage commitment. Collectively, these biological, clinical, and technical considerations strongly justify the prioritization of synovium for comprehensive molecular characterization. It is important to emphasize that the findings reported herein pertain specifically to the expression patterns and functional associations of MCDRGs within synovial tissues. Given the compartment-specific pathobiology of OA, these genes may exhibit distinct transcriptional regulation, cellular localization, and functional roles in other joint tissues, including articular cartilage and subchondral bone, where the microenvironmental cues and cell-type compositions differ substantially. Therefore, future studies should extend the validation presented herein to multiple OA-relevant tissues using matched and spatially resolved sampling strategies to determine whether MCD-associated signatures are synovium-restricted or capable of representing broader, cross-compartmental regulatory modules.

We verified the bioinformatics results through real-time quantitative reverse transcription polymerase chain reaction (RT-qPCR) detection. To verify the mRNA expression levels of the key genes, RT-qPCR experiments were conducted. A total of five groups of synovial tissues were collected from OA patients and controls each; all five sample groups were obtained from the Beijing Jishuitan Hospital Guizhou Branch and were approved by the relevant ethics review board in Guizhou Province for the use of human tissue samples (ethics approval number KT 2025020501). All patients provided written informed consent for participation in this study, and this consent was reaffirmed during the specimen collection process. Five OA patients (mean age: 64.7 ± 9.1 years; male: female = 3:2; K-L grade III-IV, n = 3; K-L grade II, n = 2) and five healthy controls (mean age: 61.2 ± 8.5 years; male: female ratio = 2:3; no history of inflammatory joint disease or OA) were enrolled. After pretreatment, the total RNA was extracted using TRIzol reagent, and purity was detected with a microphotometer. RT-qPCR experiments were performed on OA patient and control samples using a cDNA reverse transcription kit and a real-time fluorescence quantitative kit. Primers for the key genes were prepared in advance, and relative expression levels of the key genes were calculated using the 2^−△△Ct^ method. Statistical analyses were performed using GraphPad Prism 10.1.2 to calculate *p-*values at a significance level of *<* 0.05 and to generate graphical representations of the data. All experiments were independently repeated at least three times. Following RT-qPCR, the 2^−△△Ct^ values were calculated for each group from the obtained data, and the results were recorded accordingly. The data were then organized into their corresponding groups.

### 2.3 Statistical analysis

Quantitative variables are presented as the mean ± standard deviation (SD) or median (25th percentile, 75th percentile) depending on the normality of the distribution. Two-group comparisons were performed using unpaired or paired t-tests for normally distributed data; otherwise, the Mann–Whitney U test or Wilcoxon signed-rank test was applied. When comparing more than two groups, we used one-way analysis of variance (ANOVA) combined with the Tukey honestly significant difference test. For data involving multiple factors, we adopted multiway ANOVA for a more comprehensive analysis. All statistical analyses were conducted using GraphPad Prism 10.1.2 and SPSS Statistics 26.0, which were also used to generate all graphical representations. A *p*-value < 0.05 was considered to be statistically significant. Statistical analyses were also conducted using R version 4.2.0 (with the edgeR, limma, and psych packages).

## 3 Results

### 3.1 Data preparation

#### 3.1.1 Training set

The GSE89408 dataset was obtained from the GEO database (https://www.ncbi.nlm.nih.gov/); it includes synovial tissue samples from 22 OA patients and 28 normal controls. Samples derived from tissues for joint pain, undifferentiated arthritis, and rheumatoid arthritis were excluded from this study. The dataset was generated using high-throughput sequencing on the GPL11154 platform and was primarily used for differential gene expression analysis and correlation analyses of key genes.

#### 3.1.2 Validation set

The GSE55235 dataset was downloaded from the GEO database; it contains synovial tissue samples from 10 OA patients and 10 healthy controls. The synovial tissue samples associated with rheumatoid arthritis were excluded from the present study. This dataset was generated using microarray technology on the GPL96 platform and was used to validate the expression levels of the identified genes.

#### 3.1.3 MCDRGs

A total of 444 MCDRGs were retrieved from the Molecular Signatures Database (https://www.gsea-msigdb.org/gsea/msigdb/human/search.jsp) based on reference literature (Wu et al., 2024).

### 3.2 Identification and functional analysis of candidate genes

#### 3.2.1 Identification of DEGs

**FIGURE 1:**
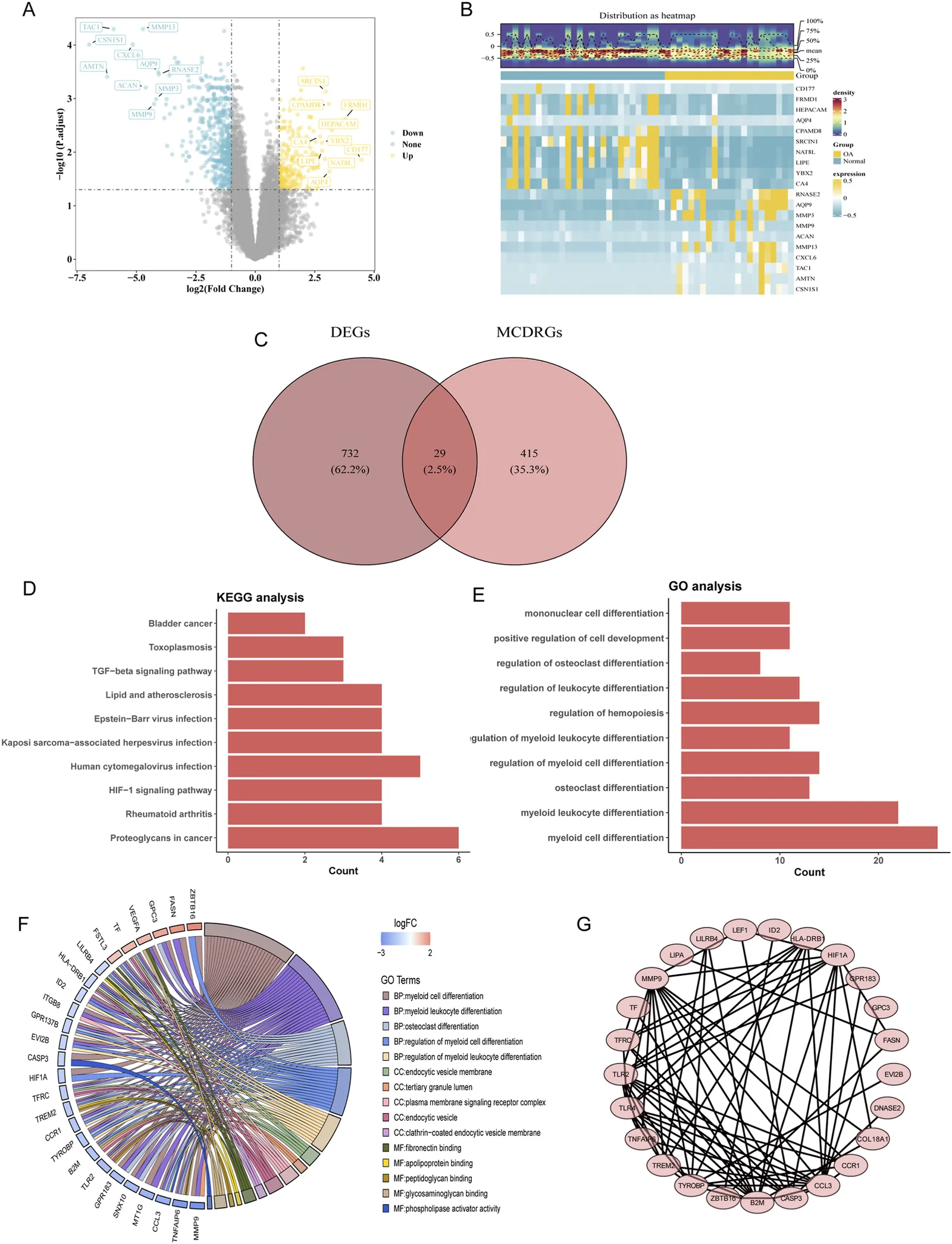
Screening and comprehensive analysis of the candidate genes: **(A)** Volcano plot of the results of the differential gene expression analysis between the osteoarthritis (OA) and normal groups in the training set. Differentially expressed genes (DEGs) were identified using thresholds of an adjusted *p* value < 0.05 and a |log_2_(FC)| > 1. The upregulated DEGs in the OA group are indicated in yellow, while the downregulated DEGs are shown in blue. The labeled genes represent the top-10 upregulated and downregulated DEGs each. **(B)** Volcano plot depicting OA versus normal patients in the training set, where the heatmap illustrates the expression patterns of the top-10 upregulated and downregulated genes. High expression levels are represented in yellow, while low expression levels are shown in blue. **(C)** Identification of candidate genes, where the Venn diagram illustrates the intersection between the DEGs and myeloid-cell-differentiation-related genes. Bar charts of **(D)** Gene Ontology (GO) and **(E)** Kyoto Encyclopedia of Genes and Genomes (KEGG) enrichment analyses; the vertical axes represent the enriched GO and KEGG pathways, while the horizontal axes indicate the number of genes associated with each of the pathways. **(F)** Chord diagram of GO enrichment analysis illustrating the distribution of genes across different GO terms and their associated pathways. **(G)** Construction of the protein–protein interaction (PPI) network, where the node size reflects centrality-based protein importance, and edge thickness corresponds to interaction confidence scores derived from integrated experimental and database evidence.

To identify the DEGs between OA patients and control samples, the R package “edgeR” (Gu et al., 2025; R Core Team, 2022) was applied to the training dataset (OA vs. control). A total of 537 upregulated and 224 downregulated DEGs were identified based on the criteria of sn adjusted *p value* < 0.05 and a |log_2_(FC)| > 1. The R package “ggplot2” (Wickham, 2016) was used to generate a volcano plot to visualize the DEGs (Figure 1A), and the top-10 upregulated and downregulated DEGs were labeled for clarity. Additionally, the expression patterns of these top DEGs were illustrated using a heatmap generated by the R package “pheatmap” (Figure 1B).

#### 3.2.2 Identification of candidate genes

To further identify the differentially expressed MCDRGs in OA patients compared to controls, the R package “ggvenn” (Yan, 2021) was employed to determine the intersection of DEGs and MCDRGs, and the results were visualized using a Venn diagram (Figure 1C). The overlapping genes were designated as the candidate genes. As illustrated in Figure 1C, among the 761 DEGs and 444 MCDRGs, 29 genes were found to be commonly expressed, and these 29 genes were defined as the candidate gene set.

#### 3.2.3 GO and KEGG enrichment analyses

To investigate the biological functions and signaling pathways associated with OA mediated by the candidate genes, GO-KEGG pathway enrichment analyses were performed using the R package clusterProfiler (Wu et al., 2021) on the identified candidate genes. The GO functional enrichment analysis was categorized into three domains under BPs, cellular components, and molecular functions. These analyses aimed to elucidate the potential roles of the candidate genes in the pathogenesis of OA (Figures 1D,E). GO enrichment analysis results (*p*-value < 0.01, *q*-value < 0.05) demonstrated that the 29 candidate genes were predominantly enriched in BPs involving MCD, myeloid leukocyte differentiation, osteoclast differentiation, regulation of MCD, regulation of myeloid leukocyte differentiation, regulation of hemopoiesis, regulation of leukocyte differentiation, regulation of osteoclast differentiation, positive regulation of cell development, and positive regulation of MCD. Results from the KEGG enrichment analysis (*p*-value < 0.05, *q*-value < 0.1) revealed that the 29 candidate genes were predominantly enriched in pathways including proteoglycans in cancer, rheumatoid arthritis, the hypoxia-inducible factor (HIF-1) signaling pathway, Kaposi-sarcoma-associated herpesvirus infection, toxoplasmosis, lipids and atherosclerosis, human cytomegalovirus infection, legionellosis, alcoholic liver disease, and inflammatory bowel disease. The chord diagram (Figure 1F) from the GO analysis results illustrates the distribution of genes across different GO terms and their associated pathways. For example, *ZBTB16* is involved in MCD and its regulation, whereas *MMP9* participates in tertiary granule lumen formation, myeloid leukocyte differentiation, and NAD^+^ nucleosidase activity.

#### 3.2.4 Construction of the PPI network

To investigate the interactions among proteins encoded by the candidate genes, the PPI network was constructed using the STRING database (http://string-db.org; Figure 1G) and visualized with Cytoscape (Smoot et al., 2011); here, each node corresponds to a protein, while each edge indicates a predicted interaction between two proteins (interaction score > 0.4). The network comprised a total of 32 nodes and 74 edges.

### 3.3 Identification and screening of key genes

#### 3.3.1 Machine learning

**FIGURE 2:**
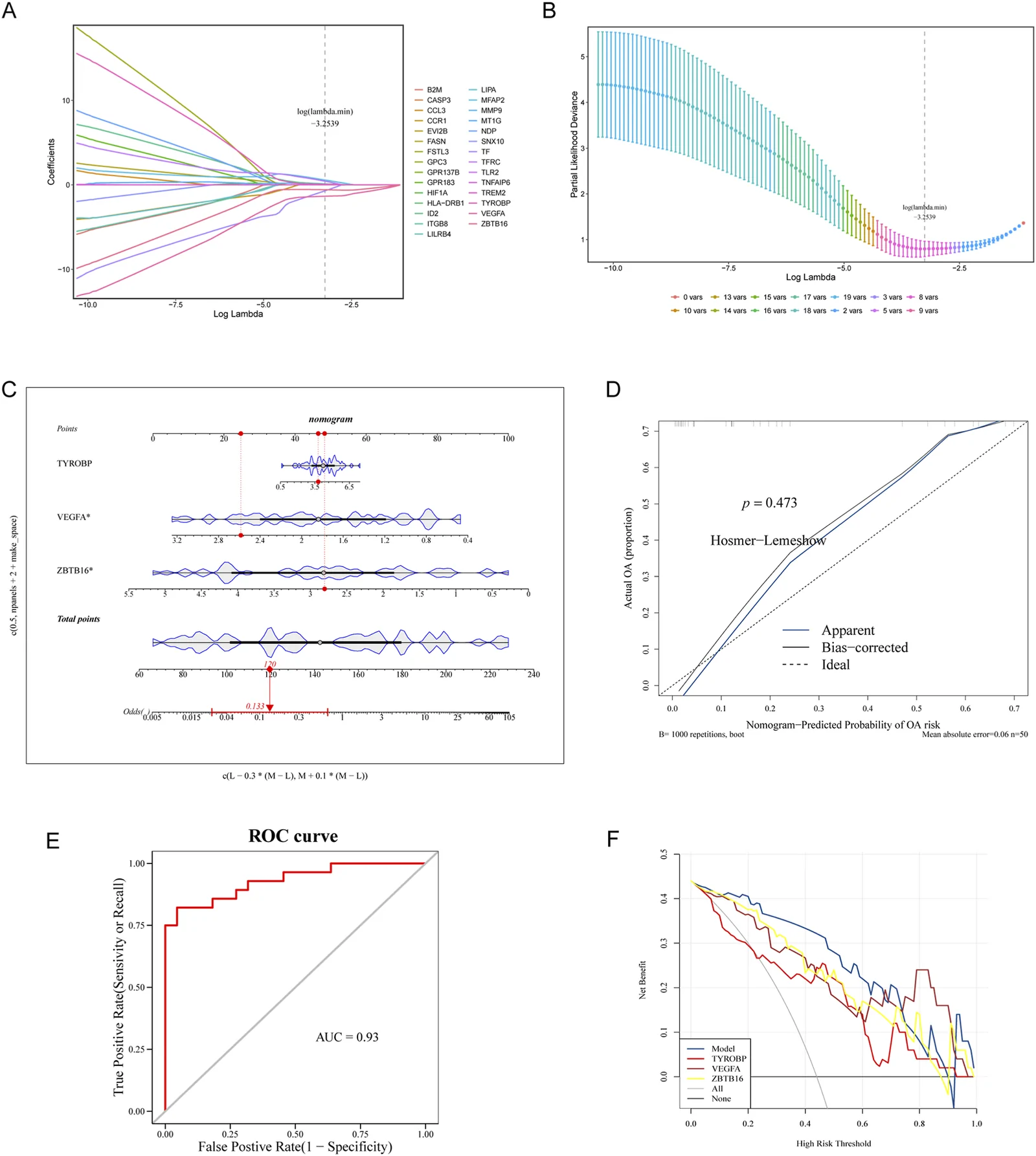
Selection of key genes. **(A)** LASSO regression algorithm: each curve in the coefficient path plot corresponds to a gene and illustrates its coefficient across different models. The x-axis displays the log-lambda values, while the y-axis represents the coefficient values for each gene. The dashed vertical line marks the log (lambda.min) value, indicating the optimal regularization parameter selected based on the minimum error. **(B)** LASSO regression algorithm: the cross-validation error curve plot displays the mean-squared error (MSE) of the model across varying lambda values. The dashed vertical line on the left indicates the value of lambda.min that yields the lowest average cross-validation error. The dashed line on the right indicates lambda.1se, which represents the largest lambda value such that the cross-validation error remains within one standard error of the minimum. Compared to lambda.min, lambda.1se results in a more regularized model that prevents overfitting while maintaining acceptable error performance. **(C)** Nomogram model of the key genes. **(D)** Calibration curve, where the horizontal axis represents the disease probability predicted by the nomogram, while the vertical axis reflects the observed (actual) disease probability. The dotted line indicates the reference line for which the predicted and observed probabilities are perfectly matched. The green solid line represents the nomogram’s predicted probabilities, while the black solid line illustrates the bias-corrected predictions obtained through bootstrapping (1,000 resamples). **(E)** Receiver operating characteristic (ROC) curve and area under the curve (AUC). **(F)** Decision curve analysis results.

To further screen the candidate genes and identify those playing critical roles in the pathogenesis of OA, the R package “glmnet” was applied to all samples in the training set. This approach employed the LASSO algorithm to shrink the coefficients of irrelevant features to zero. Candidate genes were subsequently selected based on the optimal lambda value, leading to identification of feature gene set 1 (Figures 2A,B). At lambda.min = 0.03862349, which corresponded to the model’s lowest error rate, 17 genes were identified as the feature genes; among these, eight genes with non-zero coefficients were selected as the key characteristic genes associated with knee OA: *GPR183*, *MFAP2*, *NDP*, *TF*, *TFRC*, *TYROBP*, *VEGFA*, and *ZBTB16*.

#### 3.3.2 Gene expression levels

**FIGURE 3:**
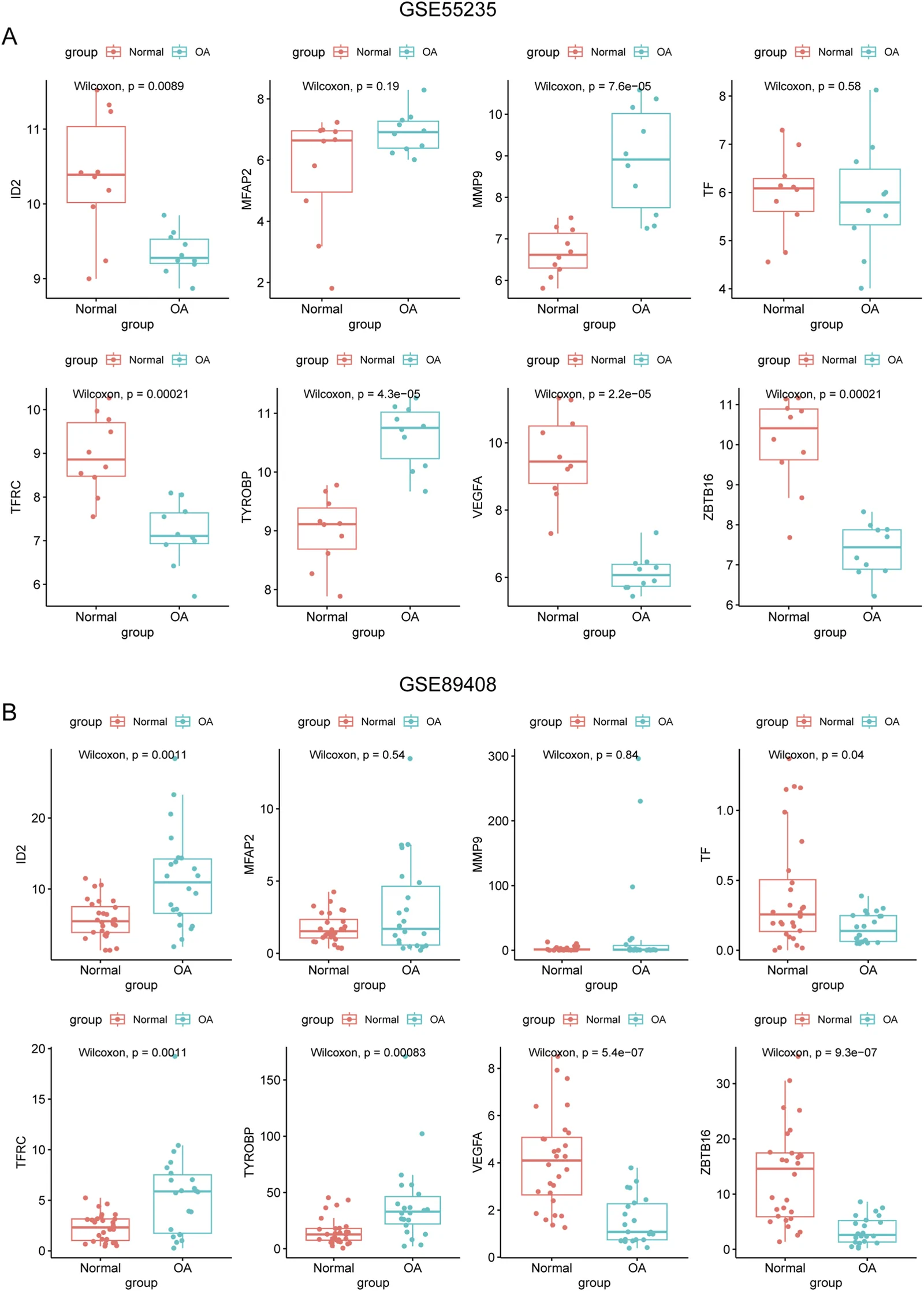
Expression levels of the characteristic genes in the OA and normal groups from the **(A)** validation set (GSE55235) and **(B)** training set (GSE89408).

To investigate the expression profiles of the characteristic genes in OA patients and control samples and to further identify the key genes, we generated box plots depicting the expression levels of the eight characteristic genes (*ID2*, *MFAP2*, *MMP9*, *TF*, *TFRC*, *TYROBP*, *VEGFA*, and *ZBTB16*) in both the training and validation sets. Genes exhibiting consistent expression patterns across both datasets and showing statistically significant differences (Wilcoxon rank-sum test, *p* value < 0.05) were selected, which resulted in three key genes: *TYROBP*, *VEGFA*, and *ZBTB16* (Figures 3A,B).

### 3.4 Construction and evaluation of biomarker-based nomograms

#### 3.4.1 Construction of a nomogram

The nomogram (also known as a nomograph) integrates multiple predictive indicators and represents them visually on a single plane using proportionally scaled line segments. This visualization enables the depiction of interrelationships among the variables within a predictive model. The underlying principle involves constructing a multivariable regression model that commonly includes Cox proportional hazards or logistic regression, where each predictor’s contribution to the outcome (determined by the magnitude of its regression coefficient) is translated into an assigned score for specific variable levels. These individual scores are then summed to yield a total score, which is converted to the probability of an outcome event through a defined functional relationship. This graphical transformation of complex regression equations enhances the interpretability of a predictive model. In the training set (GSE89408) comprising OA patients, the R package “rms” was employed to develop the nomogram. Here, each variable corresponds to a specific score (points), and the summation of these scores generates the total score (total points); this total score serves as the basis for predicting the likelihood of an individual being an OA patient (Figure 2C).

Decision curve analysis (DCA) is a widely used method for evaluating the clinical utility of a diagnostic model and its molecular markers. A key strength of DCA is its ability to incorporate the preferences of patients or clinical decision-makers into the analytical framework, thereby addressing real-world clinical decision-making needs; hence, its application in clinical research has been increasing steadily. In the present study, DCA was employed to assess the predictive values of the selected genes for clinical outcomes. Decision curves were generated for both individual gene markers and the nomogram model. Thus, four distinct models were constructed, namely three univariate models based on *TYROBP, VEGFA,* and *ZBTB16* individually along with a combined model integrating all three genes. As illustrated in Figure 2D, the decision curves of all four models remain distinctly separated from the two extreme reference lines, suggesting that these models offer clinically meaningful predictive performance.

To assess the predictive performance of the nomogram model, calibration curves are commonly employed to visually illustrate the relationships between the predicted and observed (true) probabilities. Within the calibration curve, a 45° reference line that passes through the origin and has a slope of 1 represents perfect agreement between predicted and observed outcomes. The more the plotted curve is aligned with this reference line, the higher the reliability of the model’s predictions. A key statistical measure used to evaluate model calibration is the Hosmer–Lemeshow (HL) test, which assesses discrepancies between predicted and observed outcomes. A *p*-value greater than 0.05 indicates that the model passes the HL test, suggesting no significant difference between the predicted and observed values while indicating good model calibration (Figure 2D). Conversely, a *p*-value less than 0.05 implies poor calibration with significant discrepancies between the predicted and observed outcomes. The calibration curves were generated using the R package “rms.” The curve demonstrated a slope close to 1 along with a non-significant HL test result (*p* = 0.473), indicating that the nomogram exhibits strong predictive accuracy (Figure 2E).

A receiver operating characteristic (ROC) curve is a graphical representation generated using various binary classification thresholds (cutoff values or decision thresholds) by plotting the true positive rate (sensitivity) on the vertical axis and the false positive rate (1-specificity) on the horizontal axis. The area under the curve (AUC) reflects the overall diagnostic performance, with values closer to 1 indicating higher diagnostic accuracy. Specifically, an AUC value between 0.5 and 0.7 suggests low diagnostic accuracy, while a value in the range of 0.7–0.9 indicates moderate diagnostic accuracy, and a value greater than 0.9 demonstrates high diagnostic accuracy; an AUC of 0.5 indicates no diagnostic value. To further assess the diagnostic reliability of the nomogram, the corresponding ROC curve was constructed using the R package “pROC,” and the predictive performance of the nomogram model for disease diagnosis was evaluated based on the AUC value. The AUC of the nomogram-derived ROC curve was 0.93, indicating that the nomogram model has strong predictive capability for disease diagnosis (Figure 2F).

In the independent validation cohort (GSE55235), the three-gene model (*TYROBP*, *VEGFA*, and *ZBTB16*) demonstrated excellent discriminative performance by achieving an AUC of 1.00. As shown in Supplementary Figure S1, the predicted probabilities for the OA samples were consistently higher than those for the normal controls, with no observable overlap between the two groups, indicating strong classification capability at the sample level. However, given the relatively small sample size of the validation cohort, these findings should be interpreted with caution and warrant further validation in larger and independent populations.

#### 3.4.2 Correlation analysis

**FIGURE 4:**
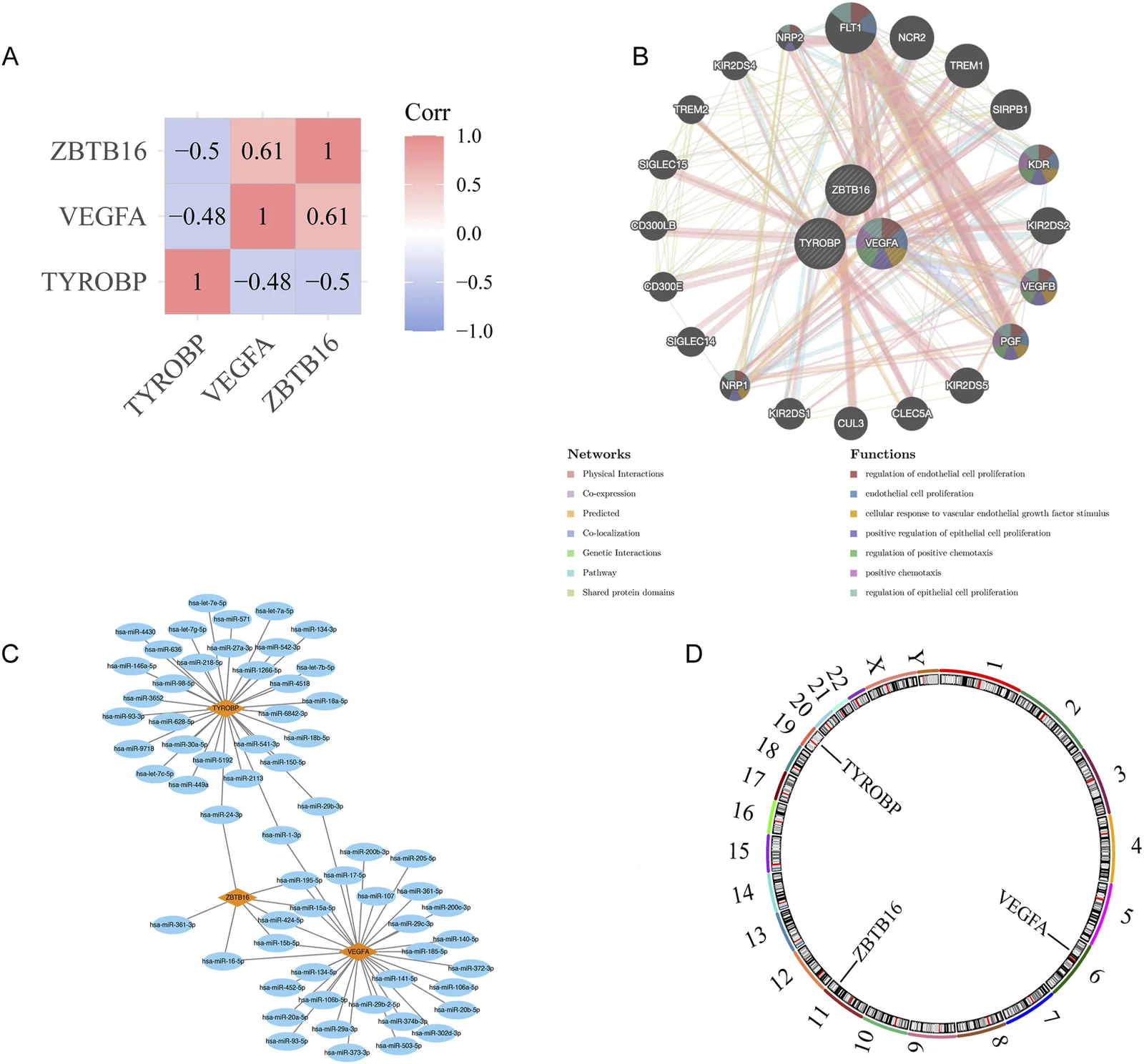
Genetic correlation analysis and network construction: **(A)** correlation analysis of the key genes; **(B)** construction of the GeneMANIA network for the key genes; **(C)** mRNA–miRNA regulatory network; **(D)** chromosome localization analysis of the key genes, where the numbers along the outer circle represent chromosomes: the numbers 1–22 correspond to human chromosomes, while X and Y represent sex chromosomes.

To investigate the correlations among the key genes, a Spearman’s rank correlation analysis was performed on all samples from the training set using the “psych” package in R; the results were visualized using the R package “ggcorrplot” (Figure 4A). The analysis revealed a moderate negative correlation between *VEGFA* and *TYROBP* (r = −0.48), a positive correlation between *VEGFA* and *ZBTB16* (r = 0.61), and a moderate negative correlation between *ZBTB16* and *TYROBP* (r = −0.50).

#### 3.4.3 Construction of the GeneMANIA network

To identify the functionally associated genes and explore the BPs and pathways involved, the GeneMANIA database (http://genemania.org) was used to predict potential functions and pathways related to the key genes. Thus, a co-expression network comprising the key genes and 20 additional functionally linked genes was constructed (Figure 4B). Functional enrichment analysis indicates that these genes are mainly involved in the regulation of epithelial cell proliferation, positive chemotaxis, cell responses stimulated by vascular endothelial growth factor, and endothelial cell proliferation.

#### 3.4.4 Construction of the miRNA–mRNA molecular regulatory network

By harnessing the convergent power of three premier miRNA target repositories (miRDB, miRTarBase, and DIANA-TarBase), we uncovered a compelling cohort of candidate miRNAs with high-confidence regulatory potential over the pivotal OA-associated biomarkers *VEGFA, ZBTB16,* and *TYROBP*. From this rigorously filtered set, a finely resolved miRNA–mRNA regulatory network was elegantly reconstructed (Figure 4C), which revealed a striking architectural duality: not only do multiple miRNAs converge upon distinct biomarker nodes, orchestrating coordinated repression across functional pathways—but each key biomarker also emerges as a nexus integrally regulated by a constellation of miRNAs. This intricate and multilayered crosstalk indicates that *VEGFA, ZBTB16,* and *TYROBP* are not merely passive markers but also central conductors in the molecular symphony of OA, i.e., dynamic hubs where regulatory signals converge, amplify, and diverge. These insights hint at a valuable and hypothesis-rich landscape for deciphering how miRNA-mediated fine-tuning sculpts the pathogenic gene expression architecture of OA.

#### 3.4.5 Chromosome and subcellular localizations

**FIGURE 5:**
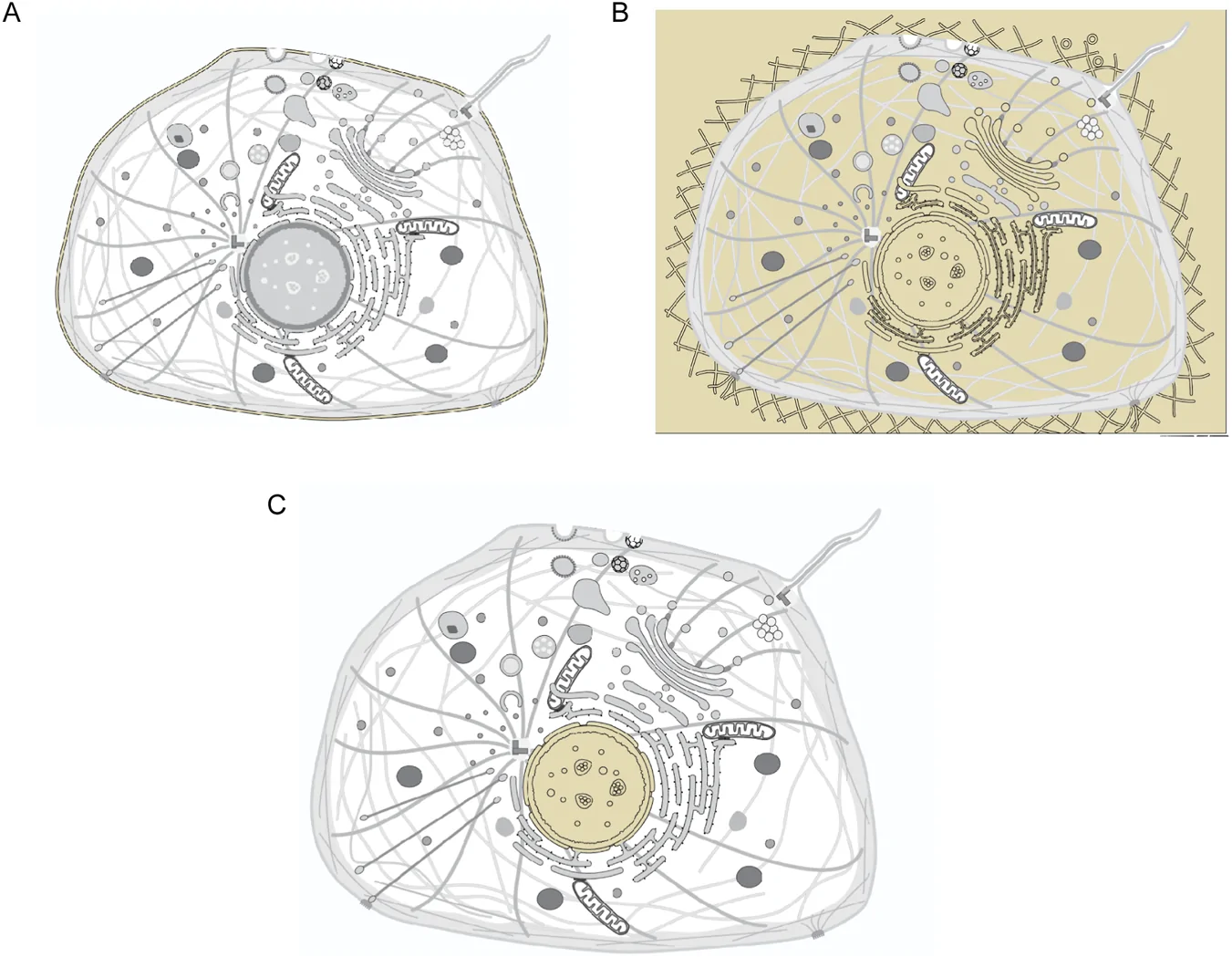
Subcellular localization of the proteins encoded by the key genes: **(A)** *TYROBP*, **(B)** *VEGFA*, and **(C)** *ZBTB16*.

To determine the chromosomal positions of the key genes in the human genome, the “OmicCircos” package was used to visualize their genomic distributions of the three key genes: *TYROBP, VEGFA,* and *ZBTB16* (Figure 4D). The results indicate that *TYROBP* is located on chromosome 19, *ZBTB16* is situated on chromosome 11, and *VEGFA* is located on chromosome 6. To elucidate the subcellular localizations, the proteins encoding these key genes provide insight into their functional roles and the BPs in which they are involved. Subcellular localization and visualization diagrams of these proteins (Figures 5A–C) were predicted using the UniProt online database (https://www.uniprot.org/). The results show that *TYROBP* is predominantly localized to the cell membrane, while *VEGFA* is distributed across multiple subcellular compartments and *ZBTB16* is primarily localized within the cell nucleus.

#### 3.4.6 Single-gene GSEA

**FIGURE 6:**
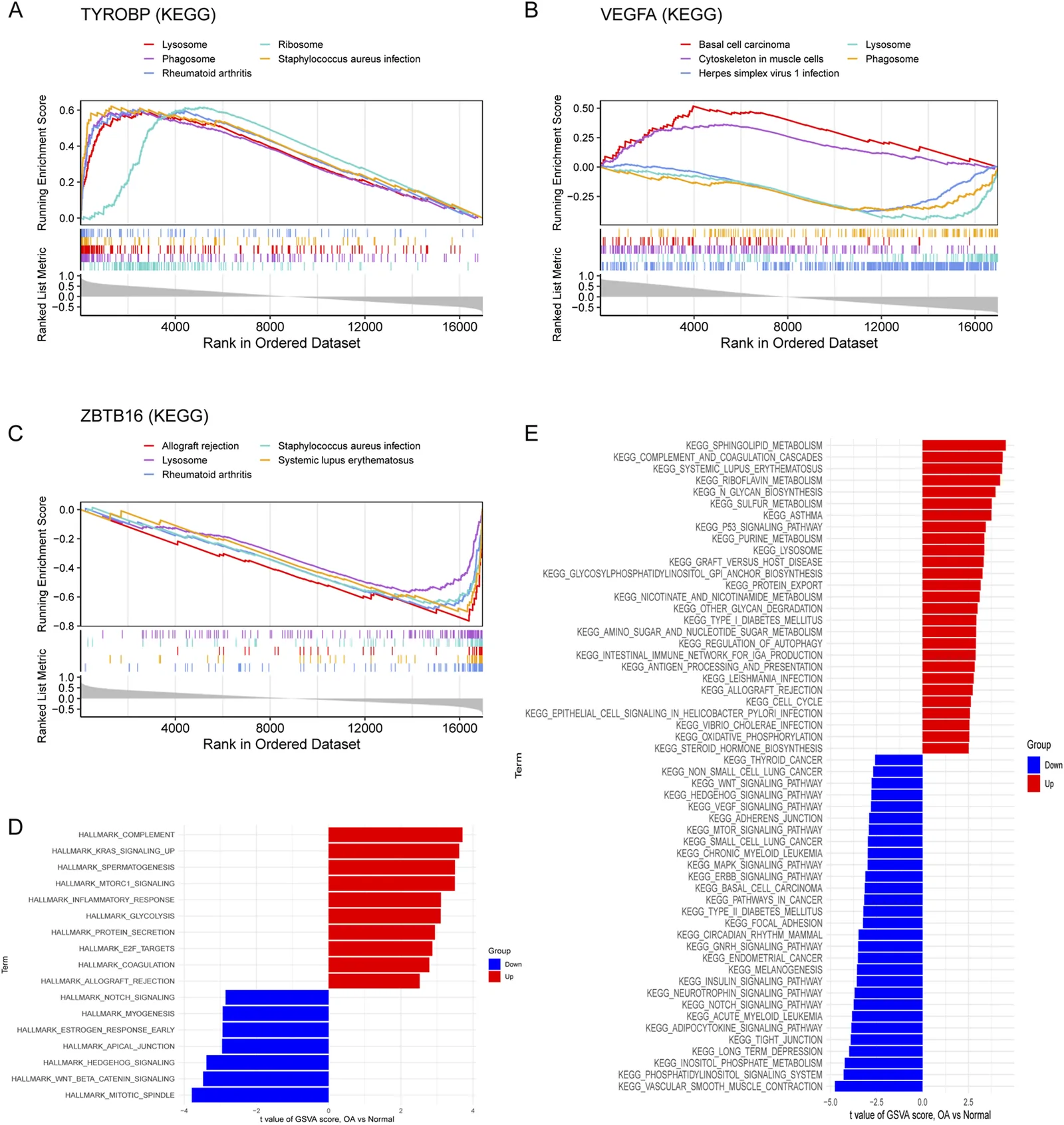
Gene-set enrichment analyses of **(A)** *TYROBP*, **(B)** *VEGFA*, and **(C)** *ZBTB16*. **(D)** Differential analysis of the gene-set verification analysis (GSVA) results for the HALLMARK pathway enrichment in the OA and control groups; here, red denotes upregulation while blue denotes downregulation. **(E)** Differential analysis of KEGG pathway enrichment by GSVA in the OA and control groups; here, red denotes upregulation while blue denotes downregulation.

To elucidate the biological functions and pathways associated with the key genes in the pathogenesis of OA, we performed a Spearman correlation analysis between each key gene and all other genes across all samples in the training set using the R package “psych” to calculate the correlation coefficients. Subsequently, the genes were ranked in descending order based on the values of these coefficients. Using this ranked gene list, we performed GSEA with the R package clusterProfiler; the reference gene set “c2.cp.kegg.v7.5.1symbols.gmt” for this analysis was obtained from MSigDB (https://www.gsea-msigdb.org/gsea/msigdb). The top-five significantly enriched pathways were visualized based on the criteria of a |NES| > 1 and an adjusted *p value* < 0.05 (Figures 6A–C). The GSEA results demonstrate that TYROBP-related genes are predominantly enriched in the pathways including lysosomes, ribosomes, phagosomes, *Staphylococcus aureus* infection, and rheumatoid arthritis; VEGFA-associated genes are primarily enriched in pathways related to basal cell carcinoma, lysosomes, the cytoskeleton in muscle cells, phagosomes, and *Herpes simplex* virus 1 infection; ZBTB16-correlated genes are significantly enriched in pathways related to allograft rejection, *S. aureus* infection, lysosomes, systemic lupus erythematosus, and rheumatoid arthritis.

#### 3.4.7 GSVA

GSVA was performed to investigate dynamic alterations in the biological pathways during the progression of OA. GSVA is a non-parametric unsupervised method for evaluating the enrichment status of gene sets; it enables the assessment of whether specific biological pathways are differentially enriched across samples by transforming the gene-level expression matrix into a gene-set-level matrix. Using all the samples from the training set, we obtained the following background gene sets from MSigDB (https://www.gsea-msigdb.org/gsea/msigdb): “Hallmark.all.v2022.1.Hs.symbols” and “c2.cp.kegg.v7.5.1.symbols.gmt.” Based on the HALLMARK and KEGG gene sets, the ssGSEA function within the R package “GSVA” was employed to calculate GSVA scores for both OA patients and control samples. Subsequently, differential pathway activity between the OA and control groups was assessed using the R package “limma” (Smoot et al., 2011), and the top-10 most enriched pathways were identified (Figures 6D,E).

GSVA is a gene-set-based enrichment approach that effectively overcomes the limitations of traditional enrichment methods in capturing subtle but coordinated changes in the minor-effect genes. This method provides a more comprehensive interpretation of the regulatory effects of functional gene modules. Unlike conventional enrichment analysis, which requires the prior identification of DEGs before performing functional enrichment and which may miss critical information due to arbitrary threshold settings, GSVA does not rely on differential gene screening; instead, it utilizes the complete gene expression profile to directly detect pathways that show significant differences between the disease and control groups.

To further investigate pathway-level differences between high- and low-expression groups of key genes, samples in the training set were stratified into high- and low-expression groups based on the expression levels of *TYROBP, VEGFA,* and *ZBTB16*. The GSVA scores were calculated for each pathway, followed by differential pathway analysis using the “limma” package to identify significant differences between the two groups. The “Hallmark.all.v2022.1.Hs.symbols” and “c2.cp.kegg.v7.5.1.symbols.gmt” gene sets from MSigDB were used as reference datasets. Pathways with an adjusted *p-value* < 0.05 and a |t| ≥ 2 were considered to be significantly enriched. Figure 6D (based on the Hallmark.all.v2022.1.Hs.symbols) displays 10 upregulated and 7 downregulated pathways, while Figure 6E (based on 2.cp.kegg.v7.5.1.symbols.gmt) shows 27 upregulated and 29 downregulated pathways. For the “Hallmark.all.v2022.1.Hs.symbols” reference set, the top-3 upregulated pathways identified by GSVA were HALLMARK_COMPLEMENT, HALLMARK_KRAS_SIGNALING_UP, and HALLMARK_SPERMATOGENESIS, while the top-3 downregulated pathways were HALLMARK_MITOTIC_SPINDLE, HALLMARK_WNT_BETA_CATENIN_SIGNALING, and HALLMARK_HEDGEHOG_SIGNALING. For the “c2.cp.kegg.v7.5.1.symbols.gmt” reference set, the top-3 upregulated pathways were KEGG_SPHINGOLIPID_METABOLISM, KEGG_COMPLEMENT_AND_COAGULATION_CASCADES, and KEGG_SYSTEMIC_LUPUS_ERYTHEMATOSUS, while the top-3 downregulated pathways were KEGG_VASCULAR_SMOOTH_MUSCLE_CONTRACTION, KEGG_PHOSPHATIDYLINOSITOL_SIGNALING_SYSTEM, and KEGG_INOSITOL_PHOSPHATE_METABOLISM.

#### 3.4.8 Immune infiltration levels (in the training set)

**FIGURE 7:**
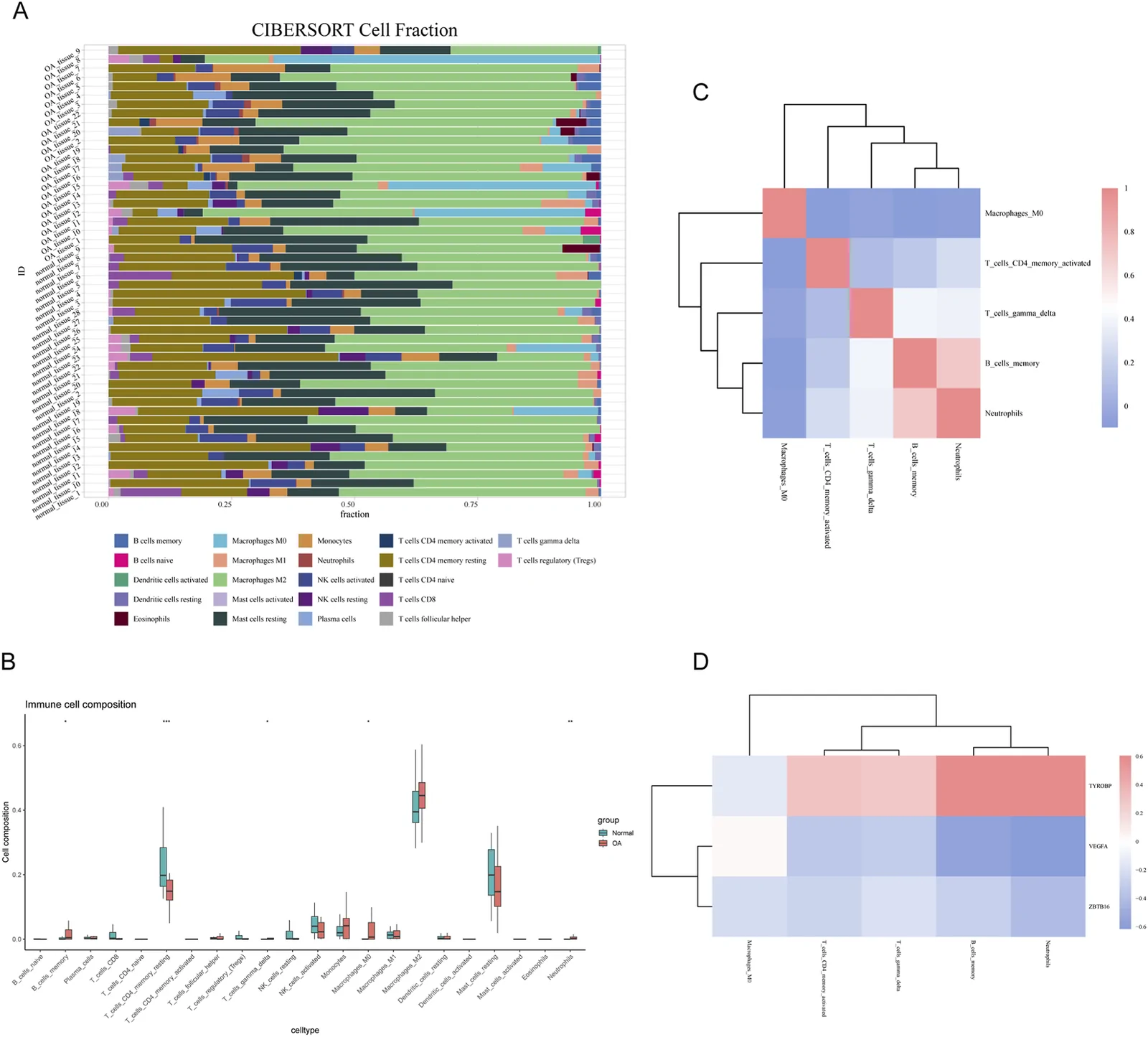
Immune infiltration and screening of differential immune cells: **(A)** distributions of immune cells in the OA patients and normal control samples; **(B)** screening of differential immune cells; **(C)** correlation analysis among different immune cells; **(D)** correlation analysis of the key genes and differential immune cells.

To further assess the differences in immune status during disease progression, the R package “IOBR” was employed to calculate infiltration scores for 22 immune cell types in the training set by comparing OA patients with the control group using the CIBERSORT algorithm. The R package “pheatmap” was used to generate a heatmap for visual representation of the immune infiltration patterns. Among all detected immune cell types, M2 macrophages, resting memory CD4+ T cells, and resting mast cells were present in the highest proportions (Figure 7A). To identify the differentially infiltrating immune cell types between the OA and control groups, we used the Wilcoxon rank-sum test to compare the immune cell infiltration levels in the training set (*p value* < 0.05), which led to the identification of significantly altered immune cells (Figure 7B). As shown in Figure 7B, the proportions of memory B cells, activated memory CD4+ T cells, gamma delta T cells, M0 macrophages, and neutrophils were significantly different between the OA and control groups.

To explore correlations among the differentially infiltrating immune cells and between the key genes and these immune cells, a Spearman correlation analysis was performed across all samples in the training set using the “psych” package in R (Figures 7C,D). The Pearson correlation coefficient (r) (Zhao et al., 2024) is a statistical measure that quantifies both the strength and direction of the linear relationship between two continuous variables; its value ranges from −1 to 1, where a positive value indicates a direct (positive) linear association, a negative value indicates an inverse (negative) linear association, and an absolute value closer to 1 reflects a stronger linear relationship. Specifically, r = 1 denotes a perfect positive linear relationship, r = −1 denotes a perfect negative linear relationship, and r = 0 indicates no linear correlation, although it does not exclude the possibility of a non-linear relationship. The correlation results revealed that the memory B cells show a weak positive correlation with the activated memory CD4+ T cells (r = 0.1468883), a moderate positive correlation with the gamma delta T cells (r = 0.3910210), a negligible negative correlation with the M0 macrophages (r = −0.0910544), and a strong positive correlation with neutrophils (r = 0.7110207). Additionally, the memory B cells exhibited a moderate positive correlation with *TYROBP* (r = 0.5992170), a moderate negative correlation with *VEGFA* (r = −0.5554077), and a weak negative correlation with *ZBTB16* (r = −0.2751086). The activated memory CD4+ T cells showed a weak positive correlation with *TYROBP* (r = 0.2975994), a weak negative correlation with *VEGFA* (r = −0.3334731), and a weak negative correlation with *ZBTB16* (r = −0.2725782). The gamma delta T cells displayed a weak positive correlation with *TYROBP* (r = 0.2836755), a weak negative correlation with *VEGFA* (r = −0.3224067), and a negligible negative correlation with *ZBTB16* (r = −0.1851311). The M0 macrophages showed a weak negative correlation with *TYROBP* (r = −0.14429759), a negligible positive correlation with *VEGFA* (r = 0.02969921), and a weak negative correlation with *ZBTB16* (r = −0.21578071). The neutrophils demonstrated a moderate positive correlation with *TYROBP* (r = 0.6045645), a moderate negative correlation with *VEGFA* (r = −0.6158295), and a moderate negative correlation with *ZBTB16* (r = −0.3971032).

#### 3.4.9 Drug prediction and *in silico* molecular docking

**FIGURE 8:**
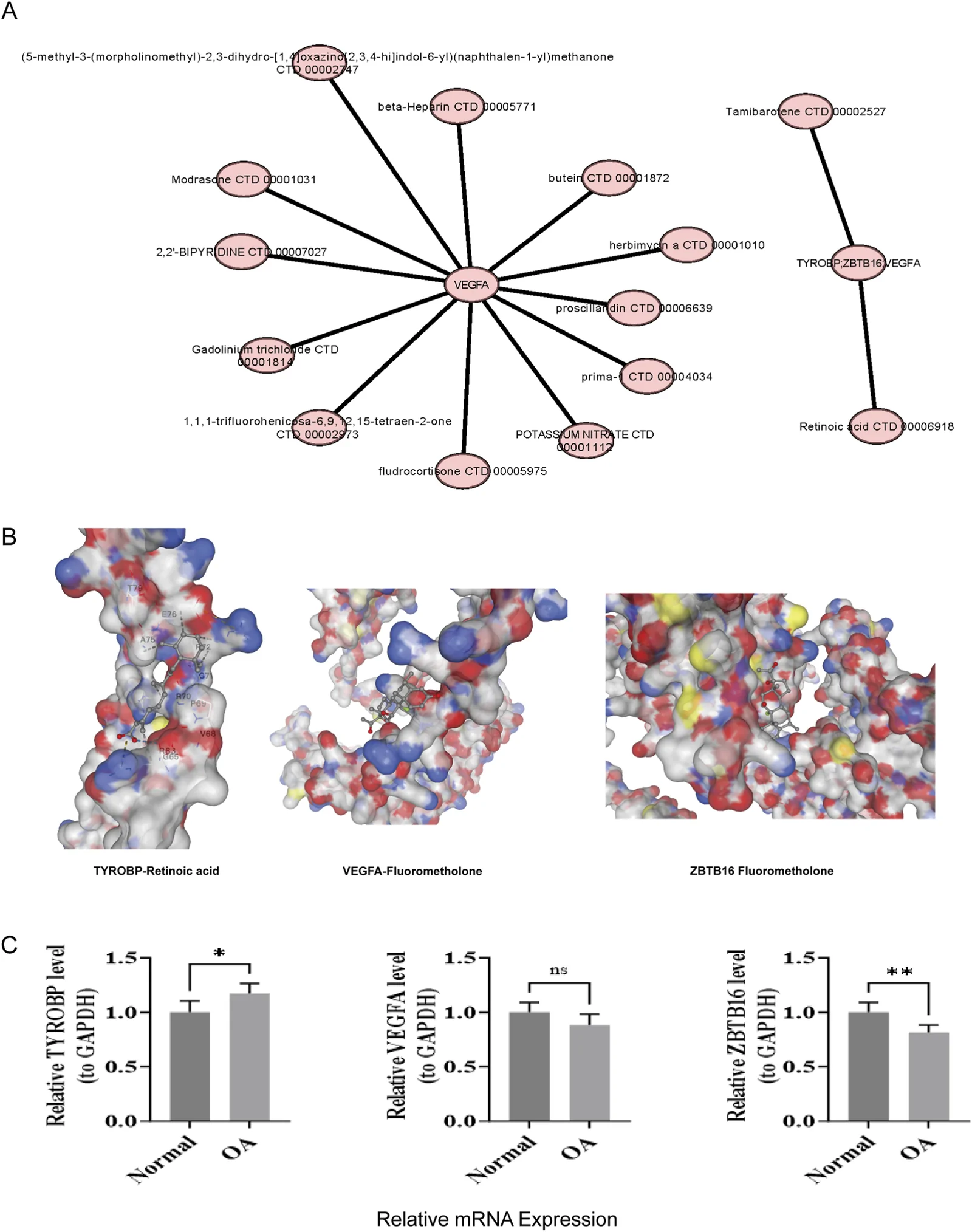
Drug prediction and molecular docking results: **(A)** network diagram for the key genes and their associated drugs; **(B)** molecular docking simulations of the key genes *TYROBP*, *VEGFA*, and *ZBTB16* showing the potential binding sites of the corresponding drug compounds. **(C)** Real-time quantitative reverse transcription polymerase chain reaction results. The synovial tissue samples were obtained from five matched pairs of OA patients and healthy controls; all donors were recruited at Guizhou Beijing Jishuitan Hospital. All experimental procedures were performed in triplicate (n > 3 independent biological replicates), and the statistical significance is denoted as follows: *p value < 0.05, **p value < 0.01, and ns (not significant) for *p* ≥ 0.05.

To identify potential small-molecule drugs with therapeutic implications for OA patients, we uploaded the identified biomarkers to the DSigDB database (https://dsigdb.tanlab.org/DSigDBv1.0/) to screen for compounds targeting these biomarkers. Candidate therapeutic agents were determined by identifying overlapping drug predictions across all biomarkers (Figure 8A). The network diagram revealed that two compounds, namely, tamibarotene and retinoic acid, exhibit potential dual regulatory effects on all three key genes: *TYROBP*, *ZBTB16*, and *VEGFA*. Subsequently, molecular docking analysis was performed between these key genes and their corresponding compounds. The three-dimensional molecular structures of fluorometholone, retinoic acid, and tamibarotene were retrieved from PubChem (https://pubchem.ncbi.nlm.nih.gov/), while the protein structures of the three key genes were obtained from the UniProt database (https://www.uniprot.org/) based on AlphaFold predictions. Molecular docking simulations were then conducted using the CB-Dock2 platform (https://cadd.labshare.cn/cb-dock2/index.php). As presented in Table 1, the lowest binding energies for *VEGFA*, *TYROBP*, and *ZBTB16* were −4.6 kcal/mol, −5.1 kcal/mol, and −5.1 kcal/mol, respectively.

**TABLE 1:** Molecular docking simulation results of key genes.

Gene	Molecule	Vina score (kcal/mol)
*VEGFA*	Fluorometholone	−4.7
*TYROBP*	Retinoic acid	−4.9
*ZBTB16*	Fluorometholone	−5.1

Subsequently, the results for the three lowest binding energy values were visualized. The visualization suggested binding sites with the lowest energies for the following pairs: *TYROBP* and retinoic acid, *VEGFA* and fluorometholone, and *ZBTB16* and fluorometholone. Structural mapping further demonstrated that these ligands could occupy the predicted binding pockets and form stable interactions with the surrounding amino residues, including hydrogen bonds and hydrophobic contacts (Figure 8B). This computational simulation is intended to be a predictive filter to identify the most promising ligand–target interactions, thereby establishing a theoretical roadmap for targeted pharmacological interrogation in the treatment of OA.

### 3.5 Experimental verification

Compared to the normal group, the RT-qPCR results demonstrated that the OA group exhibited significantly higher expression of *TYROBP* (*p* value < 0.05), lower expression of *VEGFA* without statistical significance, and significantly reduced expression of *ZBTB16* (*p* value < 0.05) (Figure 8C). The RT-qPCR-related data are provided in Supplementary Table S2. RT-qPCR validations were performed on only five paired synovial tissue samples, resulting in limited statistical power and an inability to detect modest but biologically relevant differences in *VEGFA* expression. In contrast, the GEO datasets GSE89408 (n = 50) and GSE55235 (n = 20) provide information on substantially larger multicenter cohorts encompassing a broader spectrum of OA stages from early to advanced, thereby enhancing robustness in detecting consistent transcriptional trends. Critically, the RT-qPCR cohort consisted exclusively of patients with moderate-to-severe OA (K-L grades III–IV) from a single institution, whereas the GEO samples reflect greater clinical and histopathological heterogeneity across diverse patient populations and collection protocols. These discrepancies in cohort composition, notably in terms of disease severity, anatomical sampling, and technical handling, may underlie the observed divergence in *VEGFA* expression patterns. Furthermore, transcriptomic profiling (RNA-seq or microarray) captures genome-wide expression dynamics and enables pathway-level inference, while RT-qPCR offers high sensitivity and targeted quantification of individual transcripts; the inherent differences in dynamic ranges, detection thresholds, and normalization strategies between these platforms further contribute to method-dependent variability. Therefore, independent validation of *VEGFA* expression consistency is warranted in a well-powered multicenter cohort stratified by OA stage and synovial pathology. Moreover, given the strong co-expression of *VEGFA* with the MCD core gene *ZBTB16* and the hub gene *TYROBP*, along with their collective enrichment in angiogenesis and inflammatory pathways, the functional contributions of *VEGFA* to OA synovitis likely arise from context-dependent network interactions rather than from isolated transcriptional dysregulation. Hence, mechanistic studies involving genetic perturbation and pathway-resolved functional assays are needed to dissect this cooperative regulatory logic.

## 4 Discussion

Currently, the therapeutic strategies available for OA are largely symptomatic and fail to effectively address disease progression. Increasing evidence suggests that MCD plays a critical role in regulating synovial inflammation and joint remodeling; however, its underlying molecular mechanisms in OA remain insufficiently defined. In the present study, transcriptomic and bioinformatics analyses were performed to identify three key MCD-associated genes (*TYROBP*, *ZBTB16*, and *VEGFA*) that may function as central regulators linking immune signaling, transcriptional control, and angiogenesis in OA. Previous studies have demonstrated that genes associated with MCD, including *MAGED1*, *MMP9*, *MMP13*, and *ADAMTS5*, are significantly upregulated in OA cartilage and that these genes are closely implicated in inflammatory responses, cartilage matrix degradation, and cartilage calcification (Zhao et al., 2024). Similarly, an integrative analysis of single-cell RNA sequencing and spatial transcriptomics demonstrated a marked accumulation of myeloid cells in the synovium of OA patients, with a notable expansion of “hybrid” macrophages co-expressing pro-inflammatory and anti-inflammatory markers, such as IL-10^+^/IL-1β^+^ (Li et al., 2023). These cells may originate from the perivascular fibroblast-like synoviocytes (FLSs) located in the sub-lining layer of the synovium. Pseudotemporal trajectory analysis indicates that their differentiation is governed by key transcription factors, including *KLF4*, *SOX5*, and *CREB3L2* (Choi et al., 2018). Collectively, these findings indicate that dysregulated MCD contributes to macrophage heterogeneity and inflammatory microenvironment remodeling in OA.

Machine-learning-based transcriptomic analysis was used to identify several MCD-associated genes in the present study, among which *VEGFA*, *TYROBP*, and *ZBTB16* were consistently validated across independent datasets, suggesting their potential as key regulators in OA. The expression levels of *VEGFA* in the synovial tissues of OA patients were markedly elevated compared to those in control tissues; these molecules are closely associated with the aberrant proliferation, inflammation, and angiogenesis observed in OA synovial tissues. Earlier findings have suggested that *MYC* and *VEGFA* activate the p38-MAPK signaling pathway, which subsequently enhances Jun activity, thereby promoting pathological inflammation, hyperplasia, and angiogenesis in OA synovial tissues (Luo et al., 2024; Rong et al., 2025). Accumulating evidence also indicates that *VEGFA* is critically involved in both chondrogenesis and the pathogenesis of OA. Similarly, *BCL6* and *VEGFA* are significantly upregulated in human OA cartilage and synovial tissues, and multiple independent studies have validated their combined expression as a robust predictive biomarker signature for the onset and progression of OA (Chen et al., 2023b). The non-significant RT-qPCR results for *VEGFA* likely stem from the limited sample size (n = 5) and the intrinsic regional heterogeneity of synovial tissue, wherein *VEGFA* is spatially restricted to vascularized subregions. Although bulk qPCR lacked the sensitivity to detect these localized shifts, our large-scale transcriptomic analysis robustly identified *VEGFA* upregulation. Furthermore, the strong correlation of *VEGFA* with *ZBTB16* and its enrichment in the angiogenic pathways suggest that its role in OA pathogenesis arises from coordinated network interactions rather than isolated dysregulation, highlighting the importance of functional synergy in MCD-associated synovitis.

Experimental evidence suggests that *ZBTB16* may suppress the progression of OA through transcriptional inactivation of *GRK2* (Xiong et al., 2023). Emerging evidence also suggests that increased neutrophil counts may exert protective effects against OA. The immune-related hub genes *CD4*, *CSF1R*, and *TYROBP* have been identified as promising biomarkers for OA and merit further investigation through prospective studies (Pan et al., 2025). Diagnostic analyses have been carried out to evaluate the biomarker potential of some of these genes; *TYROBP (DAP12)* is an ITAM-containing adaptor that is widely expressed in myeloid cells, where it recruits SYK and activates PI3K signaling to regulate proliferation, survival, and differentiation (Liang et al., 2021). Loss of *TYROBP* or its associated receptor, *TREM2,* has been reported to impair RANKL/M-CSF-induced osteoclast differentiation and function (Paloneva et al., 2003), thus demonstrating the essential role of *TYROBP* in myeloid lineage development (Audrain et al., 2021). In the osteoarticular system, the *TYROBP–TREM2* axis further regulates osteoclast-mediated bone resorption and remodeling (Otero et al., 2012); its deficiency is linked to skeletal abnormalities and inflammatory disorders, such as Nasu–Hakola disease, highlighting its relevance in the pathogenesis of arthritis (Satoh et al., 2012). *ZBTB16 (PLZF)* is a BTB-ZF transcription factor that regulates hematopoietic differentiation by controlling chromatin accessibility and lineage-specific transcriptional programs (Yi et al., 2025). In myeloid progenitors, it acts as a molecular switch to balance progenitor maintenance and terminal differentiation via the epigenetic regulation of developmental enhancers (Ghasemi et al., 2024). *ZBTB16* is also induced during early osteogenic commitment, and its loss impairs osteogenic gene expression and matrix mineralization, thus highlighting its essential role in skeletal lineage development (Chen et al., 2025).

Integrating GSEA with established myeloid differentiation biology shows that *TYROBP* enrichment in the lysosome and phagosome pathways suggests its role in macrophage maturation via regulation of lysosomal activity and phagocytic function, which are key features of monocyte-to-macrophage differentiation and polarization (Jung et al., 2015). In contrast, *ZBTB16,* as a transcriptional regulator of progenitor cell quiescence, was downregulated and enriched in the immune activation pathways, implying that its loss may drive aberrant myeloid differentiation and enhance inflammatory responses in OA. Together, these findings indicate that *TYROBP* and *ZBTB16* may modulate MCD through complementary mechanisms involving functional maturation and transcriptional control.

The low-frequency genetic variant *ALDH1A2*, which encodes a key enzyme in retinoic acid biosynthesis, has been associated with an increased risk of OA; furthermore, inhibition of retinoic acid signaling has been shown to attenuate cartilage degradation and synovial inflammation (Zhu and Vincent, 2023). Retinoic acid is a well-established immunomodulator that suppresses inflammatory responses and maintains immune homeostasis (Oliveira et al., 2018). In our study, molecular docking was employed as a preliminary screening tool to identify potential regulatory intersections; the analysis predicted a potential interaction between retinoic acid and TYROBP, which is a key adaptor protein mediating pro-inflammatory signaling in myeloid cells through receptors such as TREM2 and SIRPβ1 (Haure-Mirande et al., 2022). Although these *in silico* findings require rigorous experimental validation, they offer a theoretical basis for the hypothesis that retinoic acid may modulate *TYROBP*-dependent pathways in MCD. In addition, the synthetic retinoid tamibarotene showed similar docking affinity toward *TYROBP*, while fluorometholone demonstrated potential binding capabilities with *VEGFA* and *ZBTB16*, which are involved in angiogenesis and transcriptional regulation (Zhao et al., 2019; Proescholdt et al., 1999; Aajja et al., 2025; Cheng et al., 2021; Sadler et al., 2015; Kupferman and Leibowitz, 1975). These findings suggest that modulation of MCD-related signaling pathways may represent a potential therapeutic strategy, although further functional studies are required for verification.

The CIBERSORT algorithm was employed in this study to construct the immunological landscape of OA and normal tissues by estimating the proportions of 22 immune cell types. Notably, the activated memory CD4+ T cells and neutrophils displayed significant differences between the OA and control groups. Correlation analysis suggested that *TYROBP* was negatively correlated with the resting memory CD4+ T cells (r = −0.433) but positively correlated with neutrophils (r = 0.726); in contrast, *VEGFA* showed a negligible positive correlation with the resting memory CD4+ T cells (r = 0.008) and moderate negative correlation with neutrophils (r = −0.432); *ZBTB16* exhibited a weak positive correlation with resting memory CD4+ T cells (r = 0.172) and mild negative correlation with neutrophils (r = −0.239). Notably, synovial tissue heterogeneity, including fibroblast-like synoviocytes and endothelial cells, may introduce background noise in CIBERSORT analyses, while low-abundance immune subsets may be underrepresented. However, consistent patterns across independent datasets support the robustness of our results. The molecular docking results further highlight potential therapeutic targets.

Current research efforts on the relationship between OA and MCD remain limited in scope and depth. Our study presents a comprehensive and in-depth investigation of the associations between MCDRGs and OA pathogenesis. By employing multilayered bioinformatics analyses, we elucidate the intrinsic connections among inflammatory responses, immune modulation, and aberrant cartilage and bone metabolism in the context of MCD, thereby offering novel insights into the molecular mechanisms underlying OA. Although functional causality warrants further investigation, this systematic prioritization provides an essential framework for streamlining future mechanistic research toward the most biologically relevant drivers of myeloid dysregulation. Furthermore, our work provides a solid theoretical foundation and robust data support for developing innovative therapeutic strategies targeting MCD regulation. These findings hold promise for improving the quality of life for OA patients and establishing a new paradigm in OA research and clinical management. To address the inherent challenges of transcriptomic analysis, we implemented a robust multistage strategy. Cross-dataset heterogeneity was mitigated by using GSE89408 and GSE55235 as independent training and validation cohorts, respectively, thereby avoiding potential biases from data integration. To manage high-dimensional data and prevent overfitting, LASSO regression with cross-validation was employed for precise feature selection. Finally, to bridge the gap between computational predictions and biological reality, the key biomarkers (*TYROBP*, *VEGFA*, and *ZBTB16*) identified herein were validated via RT-qPCR in clinical synovial samples. This integrated approach ensures the reliability and translational relevance of our findings.

Overall, this study is based on transcriptomics data and integrates bioinformatics and machine-learning techniques to identify potential biomarkers of OA and explore their biological functions and regulatory mechanisms. Nevertheless, several limitations of this work should be acknowledged. The reliability of the findings may be influenced by data quality, algorithmic assumptions, and the inherent complexity and heterogeneity of biological systems, which may limit our ability to fully capture dynamic processes. In addition, the relatively small sample size used for RT-qPCR validation and the lack of spatially resolved sampling of synovial tissue may have affected the accuracy of gene expression assessments, particularly for genes such as *VEGFA* that exhibit spatial heterogeneity. Therefore, future studies will need to prioritize immunohistochemistry and Western blotting to map the proteomic landscape within the heterogeneous OA synovium. Furthermore, we aim to deploy loss-of-function and gain-of-function assays on primary human synovial macrophages to rigorously deconvolve the regulatory roles of *TYROBP* and *ZBTB16* in myeloid lineage commitment and their specific contributions to synovial–cartilage crosstalk. Some of the main limitations of this study are the limited sample size for RT-qPCR validation and the absence of spatially resolved sampling, specifically the failure to microdissect and separately analyze the vascularized and inflammatory subregions of the synovium. To delineate the expression patterns and functional context of *VEGFA* in OA synovitis more accurately, future studies should consider larger cohort sizes and employ region-specific molecular profiling of these histopathologically distinct compartments.

## 5 Conclusion

Integrative bioinformatics was used in this study to identify *VEGFA*, *ZBTB16*, and *TYROBP* as key regulators in OA; these genes showed consistent expression across different datasets and significant associations with neutrophil infiltration. The functional analyses showed that these genes were linked to myeloid and osteoclast differentiation, highlighting their roles in immune dysregulation. Given the imbalance of neutrophils and macrophages in OA, our findings suggest that immune cell infiltration is a central mechanism driving disease progression. Findings from molecular docking simulations further support retinoic acid and tamibarotene as potential multitarget therapeutic candidates. Collectively, the present study provides a concise mechanistic framework for MCD-driven OA and also informs some targeted therapeutic strategies.

## Data Availability

The datasets presented in this study can be found in online repositories. The names of the repositories and accession numbers can be found in the article/Supplementary Material.

## Supplementary material

SUPPLEMENTARY FIGURE S1 Diagnostic performance of the gene model for the validation set. **(A)** Receiver operating characteristic (ROC) curve analysis of the three-gene model based on the GSE55235 dataset; here, the y-axis represents the true positive rate (sensitivity), and the x-axis represents the false positive rate (1-specificity). **(B)** Box-and-whisker plot displaying the predicted probability scores across groups. Each dot represents an individual sample; the central line in each box indicates the median, while the boxes represent the interquartile ranges (IQRs).
