# Supplementary material for: Transcriptomic profiling and experimental validation of myeloid-cell-differentiation-related key genes in osteoarthritis
Source: Front Genet. 2026 Jun 17;17:1820192. doi: 10.3389/fgene.2026.1820192 (PMC13318254; doi:10.3389/fgene.2026.1820192)
Supplement: Supplementary file 2 [file DataSheet1.docx]

**The purpose of this experiment is to investigate the expression levels of immune cell signaling and regulatory factors (TYROBP), angiogenic and growth factors (VEGFA), and transcription factors with immunomodulatory functions (ZBTB16) in osteoarthritis tissues using quantitative real-time PCR (qRT-PCR).**

**September 10, 2025.Complete the collection of tissue samples for osteoarthritis：**

**The specific grouping is as follows，as illustrated in：**

| sequence number | sample name | group |
| --- | --- | --- |
| 1 | 1.1 | Normal |
| 2 | 1.2 | Normal |
| 3 | 1.3 | Normal |
| 4 | 1.4 | Normal |
| 5 | 1.5 | Normal |
| 6 | 2.1 | OA |
| 7 | 2.2 | OA |
| 8 | 2.3 | OA |
| 9 | 2.4 | OA |
| 10 | 2.5 | OA |

**September 8, 2025.Primer synthesis (human)，as illustrated in：**

| **Gene number** | **primer** | **sequence** | | **product length** |
| --- | --- | --- | --- | --- |
| NM_198125.3 | TYROBP F | TAAGTGGTCTCCGTCCTGTC | | 197 |
| NM_198125.3 | TYROBP R | AGTGATACGCTGTTTCCGGG | | 197 |
| NM_001025367.3 | VEGFA F | TCACCAAGGCCAGCACATAG |  | 151 |
| NM_001025367.3 | VEGFA R | ACAGGGACGGGATTTCTTGC |  | 151 |
| NM_006006.6 | ZBTB16 F | GAGAGGAGTTGAGGGCGATG |  | 299 |
| NM_006006.6 | ZBTB16 R | GGGTTCTGCAGCTGGATCAT |  | 299 |
| [NM_001256799.3](https://www.ncbi.nlm.nih.gov/entrez/viewer.fcgi?db=nucleotide&id=1676318038) | H-GAPDH F | ATGGGCAGCCGTTAGGAAAG |  | 135 |
| [NM_001256799.3](https://www.ncbi.nlm.nih.gov/entrez/viewer.fcgi?db=nucleotide&id=1676318038) | H-GAPDH R | AGGAAAAGCATCACCCGGAG |  | 135 |

**September 10, 2025.RNA extraction from osteoarthritis tissue，The detection of RNA concentration involves taking 1ul of RNA and adding it to a fluorescence detection instrument for concentration measurement，as illustrated in：**

| sample name | group | Ribonucleic Acid Concentration(ng/uL) |
| --- | --- | --- |
| 1.1 | Normal | 225.32 |
| 1.2 | Normal | 105.24 |
| 1.3 | Normal | 149.84 |
| 1.4 | Normal | 114.6 |
| 1.5 | Normal | 174.52 |
| 2.1 | OA | 104.96 |
| 2.2 | OA | 623.6 |
| 2.3 | OA | 251.32 |
| 2.4 | OA | 429.52 |
| 2.5 | OA | 684.64 |

**September 10, 2025: On-machine inspection**

**Collect and record the fluorescence, generate amplification curves and melting curves, and read the Ct value. The amplification conditions are as illustrated in：**

|  | **temperature** | **time** |
| --- | --- | --- |
| predegeneration | 95℃ | 2min |
| degeneration | 95℃ | 10s |
| annealing | 55℃ | 20s |
| extending | 72℃ | 30s |


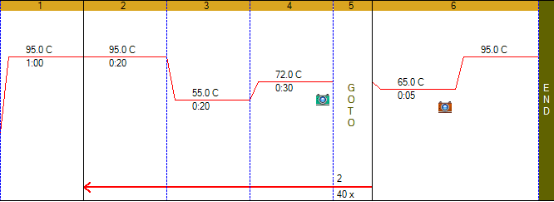


**Primer specificity exploration and TM value temperature exploration，the results are as illustrated in：**

| **Gene name** | **amplification curve** | **solubility curve** |
| --- | --- | --- |
| TYROBP | 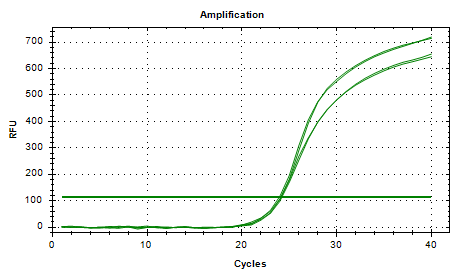 | 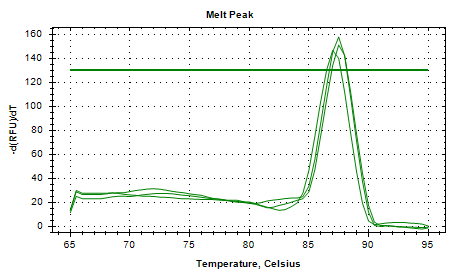 |
| VEGFA | 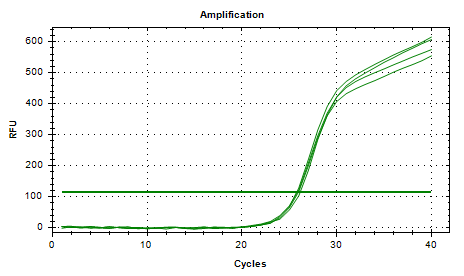 | 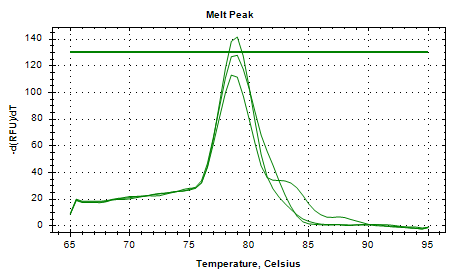 |
| ZBTB16 | 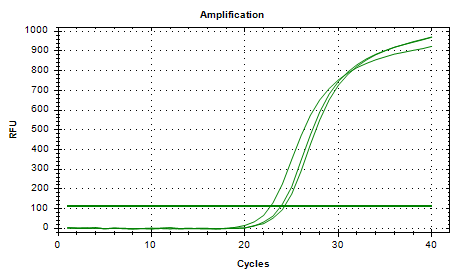 | 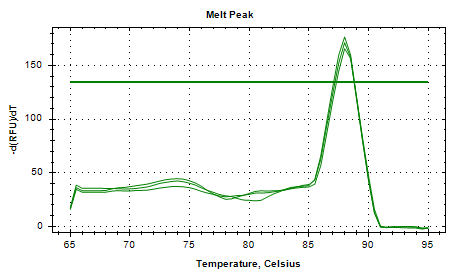 |
| GAPDH | 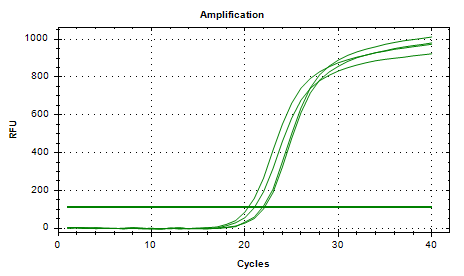 | 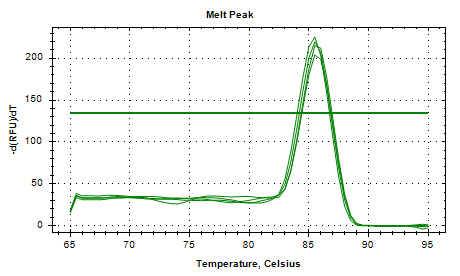 |

**Statistical analysis: All experiments were independently repeated at least three times to ensure reproducibility. Following qRT-PCR, the 2^−△△^CT method was applied to calculate relative gene expression levels from the obtained data. Results were recorded upon completion of measurements and subsequently categorized into corresponding experimental groups. Quantitative data are presented as mean±standard deviation (Mean ± SD) for normally distributed data or median (P25, P75) for non-normally distributed data, based on normality assessment. For comparisons between two groups, unpaired or paired t-tests were used for normally distributed data, while Mann-Whitney U tests or Wilcoxon signed-rank tests were employed for non-normally distributed data. Statistical analyses were conducted using GraphPad Prism 10.1.2 software, which was also used to generate all graphical representations. A two-sided P value < 0.05 was considered statistically significant. The detailed results are presented in Figure 8C：**
